# A Defective Meiotic Outcome of a Failure in Homologous Pairing and Synapsis Is Masked by Meiotic Quality Control

**DOI:** 10.1371/journal.pone.0134871

**Published:** 2015-08-06

**Authors:** Frank Mei, Peter F. Chen, Carolyn R. Dombecki, Imad Aljabban, Kentaro Nabeshima

**Affiliations:** Department of Cell and Developmental Biology, University of Michigan, Ann Arbor, Michigan, United States of America; University of North Carolina, UNITED STATES

## Abstract

Successful gamete production is ensured by meiotic quality control, a process in which germ cells that fail in bivalent chromosome formation are eliminated during meiotic prophase. To date, numerous meiotic mutants have been isolated in a variety of model organisms, using defects associated with a failure in bivalent formation as hallmarks of the mutant. Presumably, the meiotic quality control mechanism in those mutants is overwhelmed. In these mutants, all germ cells fail in bivalent formation, and a subset of cells seem to survive the elimination process and develop into gametes. It is possible that mutants that are partially defective in bivalent formation were missed in past genetic screens, because no evident meiotic defects associated with failure in bivalent formation would have been detectable. Meiotic quality control effectively eliminates most failed germ cells, leaving predominately successful ones. Here, we provide evidence supporting this possibility. The *Caenorhabditis elegans mrg-1* loss-of-function mutant does not appear to be defective in bivalent formation in diakinesis oocytes. However, defects in homologous chromosome pairing and synapsis during the preceding meiotic prophase, prerequisites for successful bivalent formation, were observed in most, but not all, germ cells. Failed bivalent formation in the oocytes became evident once meiotic quality control was abrogated in the *mrg-1* mutant. Both double-strand break repair and synapsis checkpoints are partly responsible for eliminating failed germ cells in the *mrg-1* mutant. Interestingly, removal of both checkpoint activities from the *mrg-1* mutant is not sufficient to completely suppress the increased germline apoptosis, suggesting the presence of a novel meiotic checkpoint mechanism.

## Introduction

Meiosis is a part of the developmental process that generates gametes from germ cells by halving the ploidy. During meiosis, homologous chromosomes segregate from one another in a process termed reductional chromosome segregation. Prior to segregation, homologous chromosomes recognize each other (homology recognition) and make pairs (homologous pairing) in germ cells during meiotic prophase. In most studied organisms, this initial association of homologous chromosomes is stabilized by protein structure, the synaptonemal complex (SC), and crossover resulting from meiotic recombination of homologous chromosomes. SC formation is required for crossover formation (reviewed in [1]). In this process, a pair of homologous chromosomes establishes a stable physical association termed bivalent, whereas an unpaired chromosome is called univalent. Creation of a bivalent structure is important because it ensures both proper bipolar attachment of homologous chromosome pairs to the meiotic spindle, and counteraction against the spindle pulling force until segregation begins (reviewed in [2]). Any failure in bivalent formation impairs reductional segregation, frequently causing aneuploidy in gametes. Zygotes created from aneuploid gametes often either develop into lethal embryos or contain developmental defects [3]. Successful bivalent formation in germ cells is ensured by meiotic quality control, a process involving two known meiotic checkpoint mechanisms: the synapsis checkpoint and DNA double-strand break (DSB) repair checkpoint (reviewed in [4]).

The nematode *Caenorhabditis elegans* is a model organism commonly used to study the process of bivalent formation during meiotic prophase. In *C*. *elegans*, germ cells spatiotemporally organized in the gonad contain chromosomes showing a characteristic morphological arrangement that is specific to the substages of meiotic prophase. This arrangement allows the comprehensive study of meiotic prophase and accurate identification of the substage in individual germ cells. In *C*. *elegans*, completion of SC formation (i.e., synapsis) is monitored both by PCH-2 and by a mechanism dependent on the specific chromosome locus called the pairing center (PC) [5]. If chromosomes remain unsynapsed at the PC, the synapsis checkpoint mechanism triggers apoptosis and eliminates the germ cells that are failing in bivalent formation due to incomplete synapsis. The meiotic homologous recombination process initiates with the deliberate introduction of DNA DSBs [6]. When DSBs persist without timely repair, and the meiotic homologous recombination process remains halted, germline apoptosis is triggered through the p53 homolog CEP-1 in *C*. *elegans* [7,8]. Both checkpoint mechanisms in *C*. *elegans* trigger germ cell death via apoptosis core machinery consisting of positive regulators CED-3 and CED-4, and a negative regulator CED-9 [9].

Historically, meiotic mutants in a variety of model organisms were isolated using a genetic approach that exploited a phenotype associated with gamete aneuploidy caused by the preceding failure of bivalent formation. This approach has been tremendously successful in identifying numerous essential meiosis genes [10]. In these mutants, bivalent formation is fully defective, and all germ cells fail to form proper bivalents. Since these mutants still produce some gametes (with aneuploidy), the meiotic quality control mechanisms in these mutants appear to be overwhelmed, and the organism is unable to completely remove all germ cells that have failed to form bivalents. The surviving germ cells presumably enable mutant isolation in the genetic screen. Therefore, if the number of failing germ cells is within the elimination capacity of meiotic quality control in a mutant, such a mutant is unable to be isolated as a meiotic mutant. For example, it is possible that gamete aneuploidy in a meiotic mutant that is partially defective in bivalent formation could be undetectable due to robust removal of failed germ cells via meiotic quality control mechanisms. In this report, we present evidence supporting this possibility.

We previously reported that MRG (MORF4-related gene) -1 facilitates presynaptic alignment and suppresses non-homologous SC formation during *C*. *elegans* meiosis [11]. The *mrg-1* gene encodes a conserved chromodomain-containing MRG family member [12]. In *C*. *elegans*, MRG-1 is also required for germline development as a maternal factor, germ cell proliferation and/or maintenance, and germline silencing of the *X* chromosome and transgenes [13]. It was puzzling that, while an *mrg-1* loss-of-function (*lf*) mutant did not exhibit defects in bivalent formation in most diakinesis oocytes, this mutant did exhibit defects in homologous chromosome pairing and synapsis and accumulated unrepaired DSBs in a substantial number of developing germ cells. Since we observed an increased level of germline apoptosis in the *mrg-1* mutant [11], we suspected that the germ cells that are failing in bivalent formation are eliminated by meiotic quality control. This possibility is consistent with the finding by Xu et al., which indicates that *mrg-1* RNAi induces less germline apoptosis in the mutants of meiotic quality control than in the wild type [14], although their work did not rule out the possibility that *mrg-1* RNAi is less effective for the knock down of *mrg-1* in these mutants. Here, we report that defective bivalent formation in the oocytes of the *mrg-1(lf)* mutant became evident when meiotic quality control was abrogated. Furthermore, we found that synapsis and DSB repair checkpoints are both partly responsible for removing failed germ cells in the *mrg-1* mutant. Interestingly, abrogating these two checkpoints was not sufficient to completely suppress increased germline apoptosis in the *mrg-1* mutant, suggesting the presence of a novel meiotic checkpoint mechanism.

## Results and Discussion

### *mrg-1* loss-of-function mutant fails to form bivalents in combination with germline apoptosis mutations

**Fig 1:**
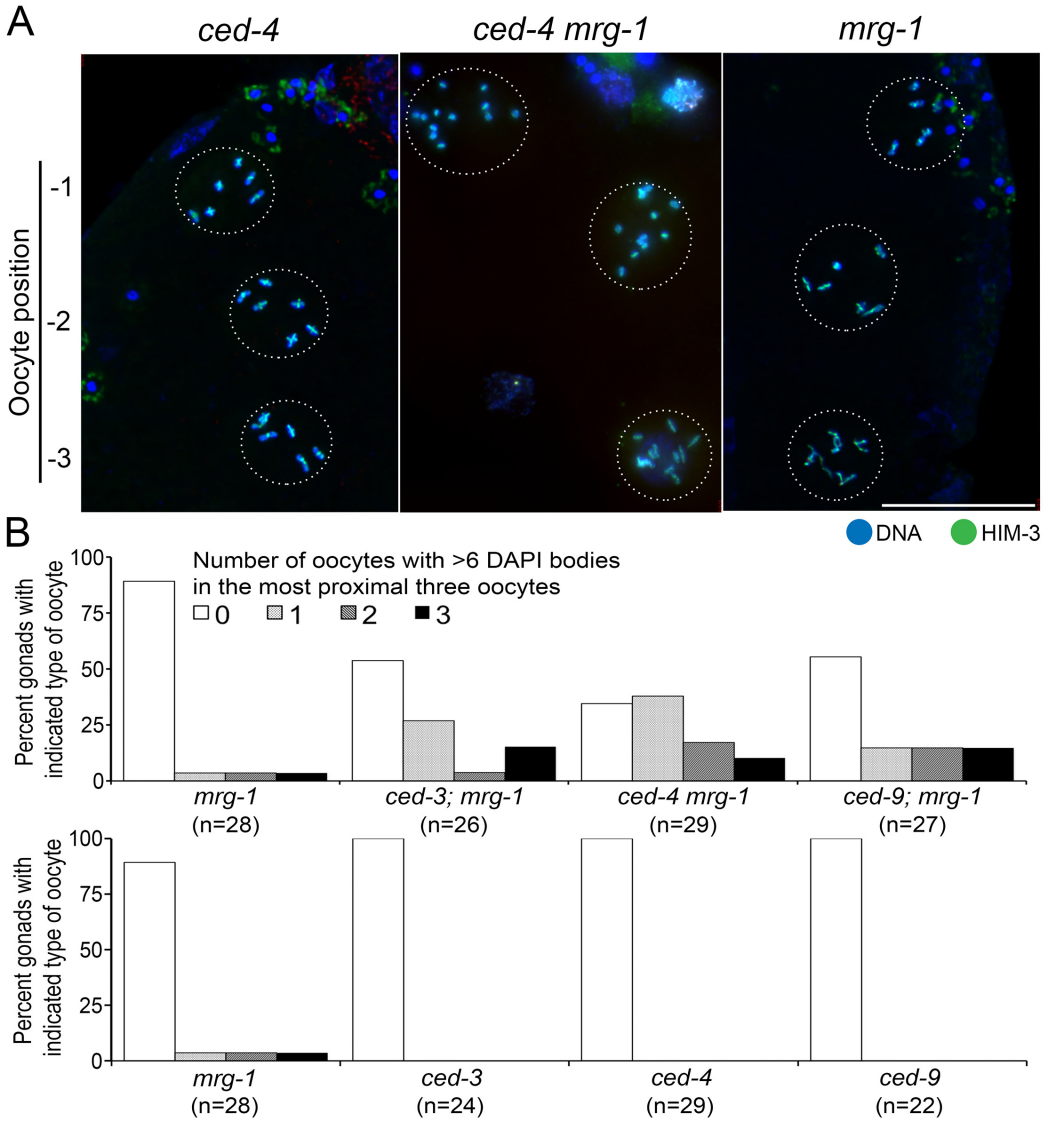
*mrg-1* (*lf*) fails to form bivalents when combined with germline apoptosis mutations. (A) Full projections of optically sectioned indirect immunofluorescence images of HIM-3 (green) with DAPI-stained DNA (blue) in oocytes from the -1 to -3 positions. The periphery of each oocyte is indicated by the dotted circle. Both the *ced-4* (left) and *mrg-1* (right) single mutants typically display six DAPI-stained bodies with cross-shaped HIM-3 staining per oocyte, demonstrating successful bivalent formation. The *ced-4 mrg-1* double mutant (middle) often displays diakinesis oocytes with more than six DAPI-stained bodies, some of which lack cross-shaped HIM-3 staining, indicating failed bivalent formation. Bar: 20 μm. (B) Histogram showing the percentage of gonads with oocytes from the -1 to -3 positions displaying more than six DAPI-stained bodies. The *ced-3; mrg-1*, *ced-4 mrg-1*, and *ced-9; mrg-1* double mutants display a substantially greater number of oocytes with more than six DAPI-stained bodies in at least one of three late diakinesis oocytes. This increase is statistically significant compared with the *mrg-1* single mutant (p = 0.0057, p<0.0001, p = 0.0067, respectively, Fisher’s exact test).

In the *mrg-1* loss-of-function mutant, failure to form bivalents in diakinesis oocytes is not obvious, while failure in homologous chromosome pairing and synapsis in germ cells during meiotic prophase is substantial [11]. Because germline apoptosis in the *mrg-1* mutant is greater than that in the wild type [11], we hypothesized that *mrg-1* mutant germ cells suffering failure in chromosome pairing and synapsis are eliminated prior to reaching diakinesis through germline apoptosis triggered by meiotic quality control. To test this hypothesis, we created double mutants using *mrg-1* and apoptosis-defective mutants *ced-3(lf)*, *ced-4(lf)*, or *ced-9(gf)*, and counted the number of 4',6-diamidino-2-phenylindole (DAPI)-stained DNA bodies in the three last oocytes (at -1, -2 and, -3 positions). If our hypothesis is correct, we should observe reduced bivalent formation in the double mutants. Because *C*. *elegans* carries 12 chromosomes (2n = 12), observing more than six DAPI-stained bodies in diakinesis indicates the presence of univalent chromosomes, and a possible failure in bivalent formation. While we have previously reported that such failed oocytes are rarely seen in the *mrg-1* single mutant, about 44–66% of double mutant worms exhibited more than six DAPI-stained bodies in at least one of three late diakinesis oocytes (Fig 1). This result indicates that germ cells that become diakinesis oocytes with failed bivalent formation (most likely due to failed homologous pairing and synapsis) in the *mrg-1* mutant are eliminated by germline apoptosis and do not reach diakinesis.

### *mrg-1* loss-of-function mutant exhibits increased synapsis failure when combined with germline apoptosis mutations

**Fig 2:**
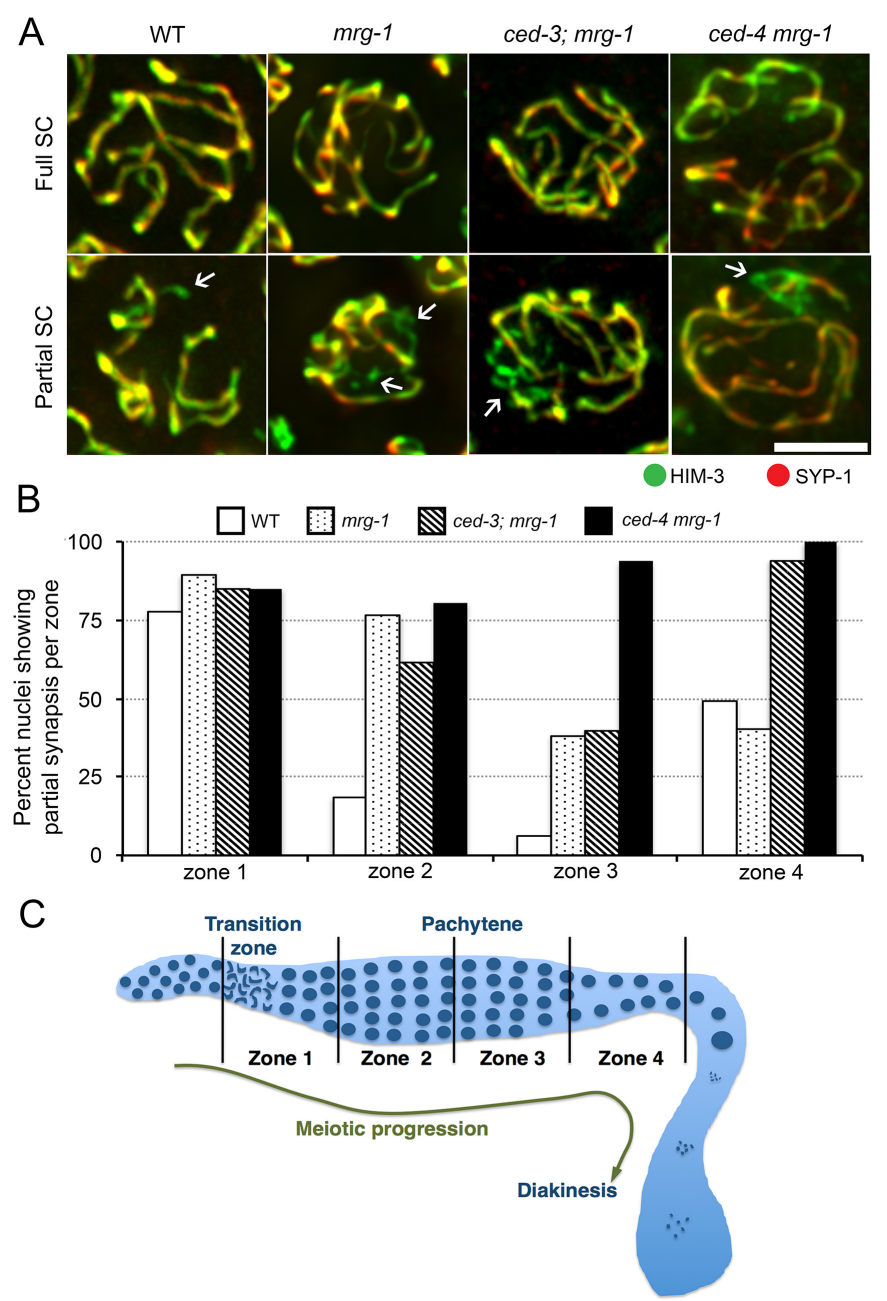
*mrg-1* (*lf*) exhibits increased synapsis failure when combined with germline apoptosis mutations. (A) Full projections of optically sectioned indirect immunofluorescence images of HIM-3 (green) and SYP-1 (red) in pachytene nuclei. Examples of nuclei that were scored as having partial SC are indicated with arrows (see Materials and Methods for a description of the scoring method). Bar: 5 μm. (B) Quantification of SC assembly. The histogram shows the percentage of nuclei with partial synapsis in each of the four subzones. (C) Schematic diagram of germline subzones used to score partial synapsis.

We previously reported that the *mrg-1* mutant exhibits nuclei with incomplete synapsis in the early to middle pachytene stage. If germ cells that fail in synapsis are eliminated by germline apoptosis, the introduction of germline apoptosis mutations into the *mrg-1* mutant might be expected to further increase the number of cells that exhibit such a synapsis defect. We examined this possibility by observing loading of the meiotic chromosome axis protein HIM-3 [15] and the SC central region protein SYP-1 [16] onto chromosomes. As shown in Fig 2, combination of a *ced-4* mutant with the *mrg-1* mutant further increased the number of nuclei showing partial loading of SYP-1 indicating partial synapsis in the middle and late pachytene stages. Thus, nuclei that fail to complete synapsis in the *mrg-1* mutant are indeed eliminated by germline apoptosis at a timeframe beginning with the middle pachytene stage. Additionally, when *ced-3* and *mrg-1* mutants were combined, the number of nuclei with partial synapsis in the late pachytene stage increased. These results again support our hypothesis. However, we did not observe more nuclei with partial synapsis in the middle pachytene stage in the *ced-3; mrg-1* double mutant than in the *mrg-1* mutant alone. Failure to complete synapsis in the *ced-3; mrg-1* mutant was less than in the *ced-4 mrg-1* mutant, possibly due to the presence of a *ced-3*-independent pathway for germline apoptosis, as previously proposed for the *meDf2/+* strain [5]. Although *ced*-4 is upstream of *ced-3*, overexpression of *ced-4* in the anterior lateral microtubule neurons induces death of these neurons, which is not completely reversed by blocking *ced-3* [17]. This and other findings support the presence of a *ced-3* independent pathway for apoptosis induction (reviewed in [18]). The presence of a *ced-3* independent apoptosis pathway downstream of *ced-4* in germline apoptosis could also explain the reduced failure of bivalent formation in the *ced-3; mrg-1* mutant than in the *ced-4 mrg-1* mutant (Fig 1).

### DNA damage and synapsis checkpoints are partially responsible for increased germline apoptosis in *mrg-1*

**Fig 3:**
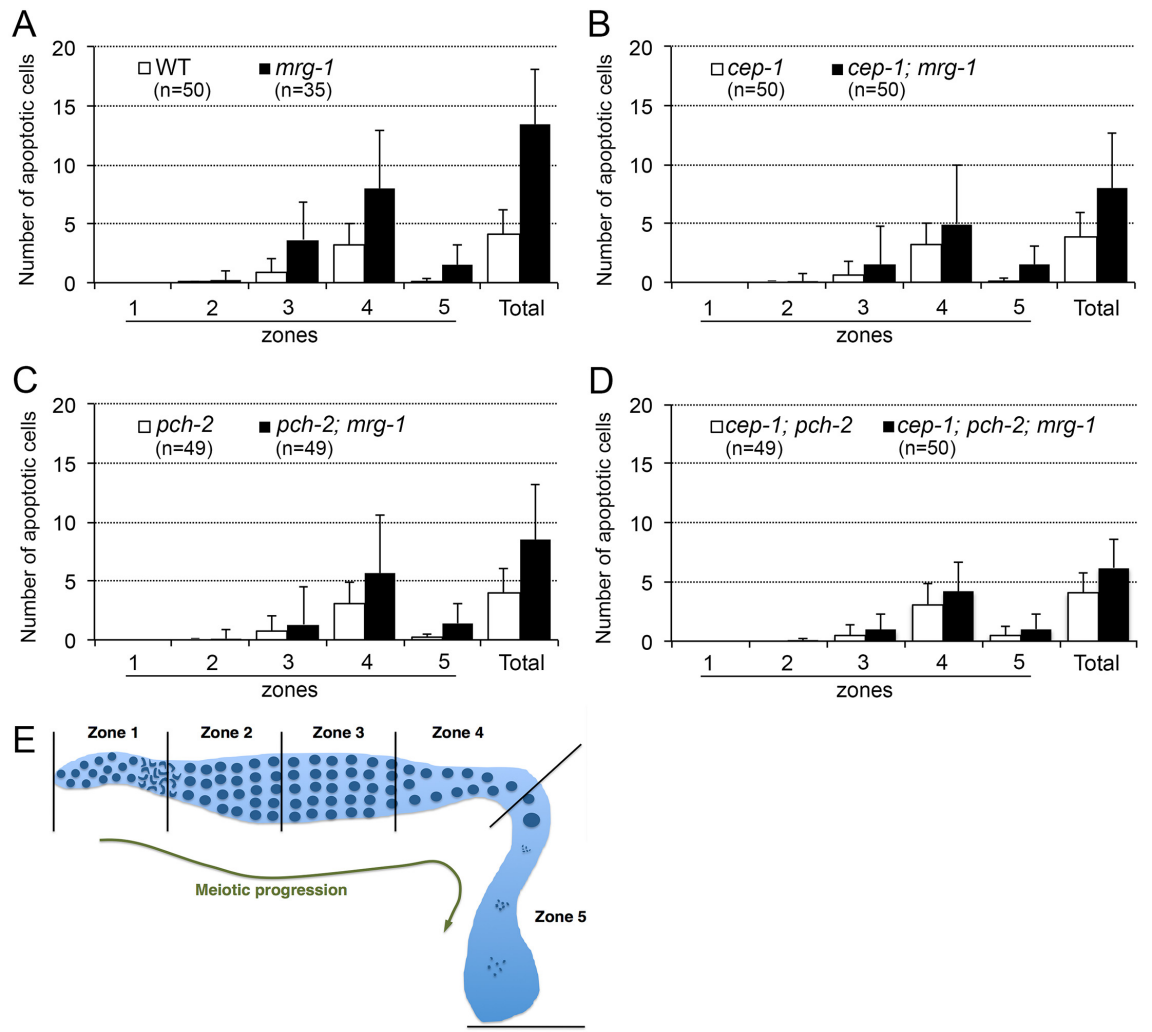
Induction of germline apoptosis in *mrg-1(lf)* is partly dependent on *cep-1* and *pch-2*. Histograms comparing wild-type and *mrg-1* mutant worms (A); *cep-1* and *mrg-1;cep-1* mutants (B); *pch-2* and *mrg-1; pch-2* mutants (C); and *cep-1; pch-2* and *cep-1; pch-2; mrg-1* mutants (D). Schematic diagram of germline subzones used to score apoptotic cell corpses (E). The *cep-1; mrg-1* mutant showed fewer total apoptotic cells than did the *mrg-1* mutant (p<0.0001, Mann-Whitney test). However, this number is still significantly higher than that seen with the wild type or the *cep-1* single mutant (p<0.0001). Similarly, the *pch-2; mrg-1* mutant showed fewer apoptotic cells than did the *mrg-1* mutant (p<0.0001), but more apoptotic cells than either the wild type or the *pch-2* single mutant (p<0.0001). The *cep-1; pch-2; mrg-1* triple mutant showed fewer apoptotic cells than the *mrg-1* single mutant (p<0.0001), *cep-1; mrg-1* double mutant (p = 0.0214), and *pch-2; mrg-1* double mutant (p = 0.0001), but significantly more than either the wild type or the cep-1; pch-2 double mutant (p<0.0001). The standard error bars indicate 1 standard deviation.

Two known checkpoint mechanisms for meiotic quality control include a DNA DSB repair checkpoint that monitors the status of meiotic DSB repair, and a synapsis checkpoint that monitors the status of SC central region formation [4]. The observed increase in germline apoptosis in *mrg-1* mutants is assumed to be triggered by the DNA damage checkpoint because *mrg-1* mutants accumulate RAD-51 foci, indicating an accumulation of unrepaired DSBs [11]. Although the *mrg-1* mutant exhibits a synapsis defect, it undergoes successful homologous pairing and synapsis at the PCs. Thus, activation of the synapsis checkpoint is not expected. Rather, homologous pairing and synapsis defects are limited to non-PC parts of the chromosomes in this mutant [11]. We examined which meiotic checkpoint is responsible for increased germline apoptosis in the *mrg-1* mutant using a genetics-based approach. The *mrg-1* single mutant was combined with the *cep-1* and/or *pch-2* loss-of-function mutants, which display defective DSB repair and synapsis checkpoints, respectively. Germline apoptosis in these mutants was monitored by observing CED-1::green fluorescent protein (GFP) accumulation around apoptotic cell corpses [19] relative to meiotic prophase progression. As we reported previously, we observed more apoptotic corpses during meiotic prophase in the *mrg-1* single mutant than in the wild type (Fig 3A). When a *cep-1* loss-of-function mutation was combined with the *mrg-1* mutant, the number of apoptotic corpses decreased; however, this mutant still displayed more apoptotic cell corpses than did wild-type worms (Fig 3B). This result indicates that the DSB repair checkpoint is partially responsible for inducing germline apoptosis in the *mrg-1* mutant and that mechanisms other than DSB repair checkpoint are involved. When a *pch-2* loss-of-mutation was combined with the *mrg-1* mutant, the decrease in apoptotic cells was similar to that in a *cep-1; mrg-1* double mutant (Fig 3C) indicating that the synapsis checkpoint is also responsible for activating germline apoptosis in the *mrg-1* mutant. This result contrasts with expectations based on previous studies, and indicates that the synapsis checkpoint can be activated even when only non-PC chromosome parts fail to synapse and the PCs still undergo successful homologous synapsis.

Because the DNA damage and synapsis checkpoints are the only two checkpoints that have been reported to trigger germline apoptosis to date, we asked whether abrogating both of these processes would completely eliminate the increased germline apoptosis in the *mrg-1* mutant. For this, we made a *cep-1; pch-2; mrg-1* triple mutant and found fewer apoptotic cells in the triple mutant than in either the *cep-1; mrg-1* or *pch-2; mrg-1* double mutants. Surprisingly, the number of apoptotic corpses in the triple mutant was still greater than that seen in wild-type worms and in the control double mutant *cep-1; pch-2* (Fig 3D). The difference was extremely significant (both p<0.0001, Mann-Whitney test) suggesting that checkpoints other than the DSB repair and synapsis checkpoints trigger germline apoptosis in the *mrg-1* mutant.

Our observation is the first demonstration that *cep-1* and *pch-2* are responsible for inducing germline apoptosis in the *mrg-1* mutant. Of note, Xu et al. previously suggested this possibility based on their observation that *cep-1*, *pch-2*, or *cep-1; pch-2* mutant worms fed with the *mrg-1* RNAi bacterial clone displayed higher levels of apoptosis than those in worms fed with the control RNAi mock clone, but lower levels of apoptosis than those in the wild type. RNAi is well-known for variable penetrance and expressivity [20]. Induction of the phenotype (i.e., induction of the full level of apoptosis in this case) is a reliable indicator of effective RNAi, but it is more difficult to draw conclusions from a negative RNAi result (i.e., no or less induction of apoptosis in this case) [21]. They evaluated the efficacy of *mrg-1* RNAi by the loss-of-germline phenotype in a certain population of the progeny of the worms that were fed with *mrg-1* RNAi bacterial clones. *mrg-1(lf)* homozygous mutant is known to exhibit a maternal effect sterile phenotype [13]: The F1 generation of the homozygous *mrg-1* mutant that is produced by heterozygous hermaphrodites (i.e., P0 generation) properly develop the germline, but the F2 generation of homozygous worms is defective in germline development and lacks a germline. Therefore, *mrg-1* RNAi was fully effective in these worms lacking a germline. In the remaining worms, however, *mrg-1* RNAi is less effective to an unknown extent. The problem is that those remaining worms were used to analyze germline apoptosis. Thus, the result presented by Xu et al. equally supports the possibility that *mrg-1* RNAi was less effective in *pch-2*, *cep-1*, and *cep-1; pch-2* mutants, and induces less apoptosis than that in the wild type. We found that combining both *cep-1* and *pch-2* with the *mrg-1* mutant is not sufficient to completely suppress the increased apoptosis, whereas Xu et al. did not observe such a residual increase [14]. The differences between these two studies strongly support the possibility that *mrg-1* RNAi was less effective in the *cep-1; pch-2* double mutant than in the wild type. Theoretically, it is possible that an unknown hypothetical additional mutation(s) in the original *mrg-1(qa6200)* strain, XA6226, is responsible for this remaining apoptosis induction. However, this possibility is extremely unlikely, considering the extensive genetic backcross (ten times) of the original strain, as well as additional genetic crosses during the production of the triple mutants.

### Non-homologous chromosome interaction itself does not seem to trigger germline apoptosis

**Fig 4:**
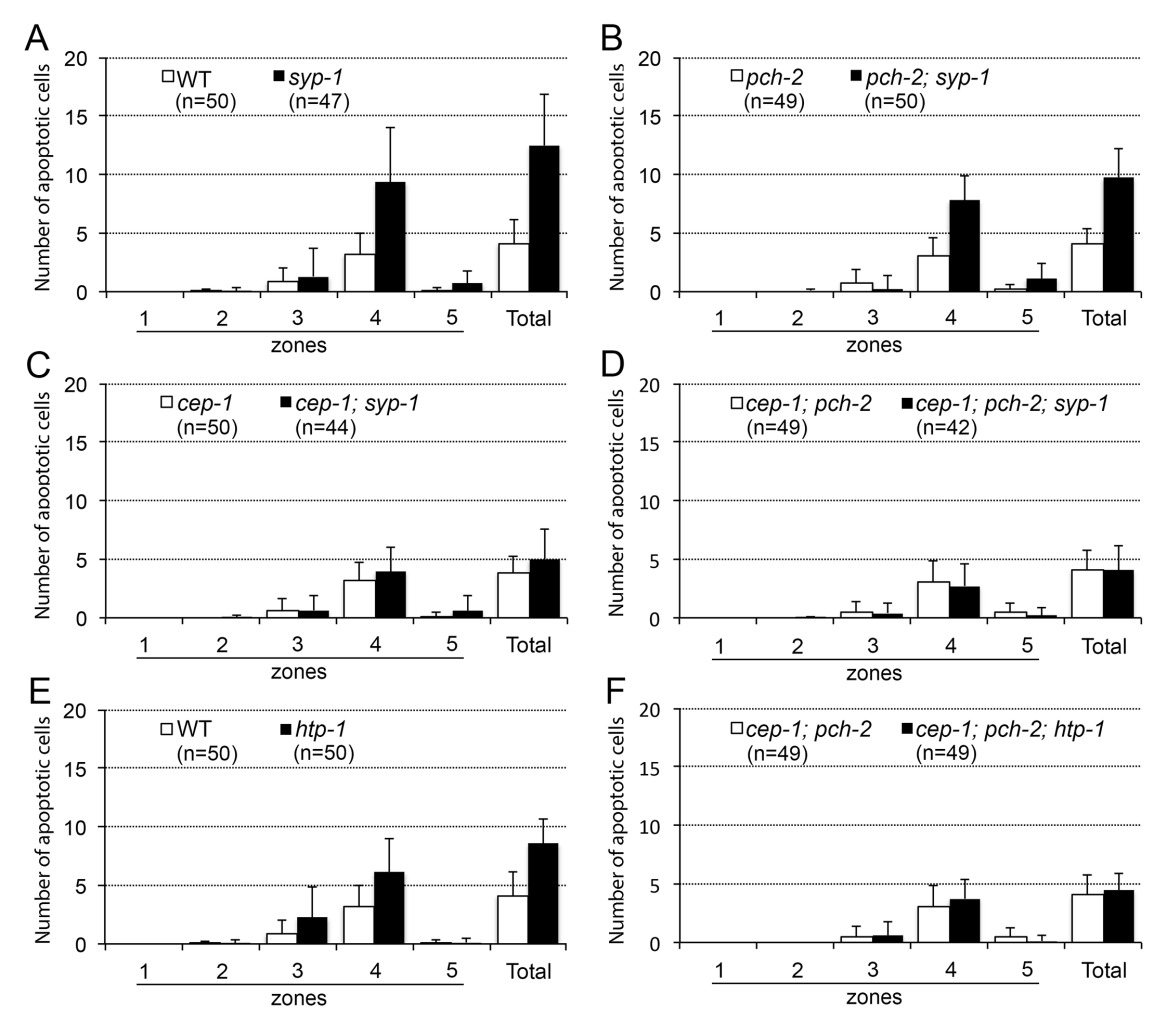
Induction of germline apoptosis depends on *cep-1* and *pch-2* in *syp-1(lf)* or *htp-1(lf)*. Histograms comparing wild-type worms with the *syp-1* mutant (A); *pch-2* and *pch-2; syp-1* mutants (B); *cep-1* and *cep-1; syp-1* mutants (C); *cep-1; pch-2* and *cep-1; pch-2; syp-1* mutants (D); wild-type worms and *htp-1* mutant (E); and *cep-1; pch-2* and *cep-1; pch-2; htp-1* mutants (F). The *pch-2; syp-1* mutant had significantly fewer apoptotic cells than did the *syp-1* mutant (p = 0.0016, Mann-Whitney test). However, this number was still significantly higher than observed in wild-type worms or in the *pch-2* single mutant (p<0.0001). The *cep-1; syp-1* mutant also had significantly fewer apoptotic cells than did the *syp-1* mutant (p<0.0001), and significantly more than did *cep-1* single mutants (p<0.05). The *cep-1; pch-2; syp-1* triple mutant had fewer apoptotic cells than the *syp-1* mutant, *cep-1; syp-1* double mutant, and *pch-2; syp-1* double mutant. This decrease is statistically significant except for *pch-2; syp-1* compared with the *cep-1; pch-2; syp-1* triple mutant (p<0.0001, p = 0.1065, p<0.0001, respectively). However, the number of apoptotic cells for the *cep-1; pch-2; syp-1* triple mutant was not significantly higher than that for the wild type or the *cep-1; pch-2* double mutant (p = 0.8979 or p = 0.9760, respectively). The *cep-1; pch-2*, *htp-1* triple mutant had significantly fewer apoptotic cells than did the *htp-1* mutant (p<0.0001). The level of apoptosis in the *cep-1; pch-2*, *htp-1* triple mutant was similar to that in the wild type, and was not significantly different from the wild type or *cep-1; pch-2* mutant (p = 0.2655 or p = 0.2006, respectively). The standard error bars indicate 1 standard deviation. WT data in 3A were reused for 4A and 4E. *pch-2* data in 3C were reused for 4B. *cep-1* data in 3B were reused for 4C. *cep-1; pch-2* data in 3D were reused for 4D and 4F.

Introduction of the *pch-2* and *cep-1* mutations eliminated increased germline apoptosis in the synapsis-defective mutant *syp-1*, a loss-of-function mutant (defective in both synapsis and DSB repair [16,22]). Thus, the remaining increase in germline apoptosis in the *cep-1; pch-2; mrg-1* triple mutant was not due to irregularity of these mutants (Fig 4A). It is possible that an unknown meiotic checkpoint mechanism triggered germline apoptosis in the *mrg-1* mutant, and this checkpoint could have caused an increase in apoptotic corpses even in the absence of DSB repair and synapsis checkpoint activities. One unique meiotic defect in the *mrg-1* mutant is SC formation among non-homologous chromosomes [11]. This improper interaction among non-homologous chromosomes might have triggered germline apoptosis through an unknown mechanism that is independent of DSB repair or synapsis checkpoints. We explored this possibility by observing germline apoptosis in the *htp-1* mutant, which shares the phenotype of non-homologous synapsis with the *mrg-1* mutant. Previous reports showed that the *htp-1* mutant exhibits severe non-homologous synapsis among autosomes in most germ cells [23,24]. If there is a checkpoint mechanism that monitors non-homologous association independently of the DSB repair and synapsis checkpoints, a greater number of germline corpses would be present in the *cep-1; pch-2; htp-1* triple mutant than in the wild type. However, as shown in Fig 4F, the absence of both of DSB repair and synapsis checkpoints (conditions present in the *cep-1; pch-2; htp-1* triple mutant) resulted in no significant increase in the number of germ cell corpses over the wild type. This result suggests that non-homologous chromosome interaction itself does not trigger germline apoptosis in the *mrg-1* mutant. However, our data does not completely rule out this possibility, because *htp-1* itself might function in such a checkpoint (i.e., monitoring chromosome interaction) and the *htp-1* mutant might be defective in this hypothetical checkpoint pathway. One possible mechanism that might cause increased germline apoptosis in the absence of known meiotic checkpoint functions is an increase in the total number of oocytes. However, this is unlikely because the number of germ cells is reduced by about 50% in the *mrg-1* mutant [11]. Another possible cause is increased apoptosis that does not contribute to meiotic quality control (i.e., physiological apoptosis). In this case, failure to form bivalents in *cep-1; pch-2; mrg-1* (with two known defective meiotic checkpoints) should produce the same level of failure in *ced-4 mrg-1* (where no germline apoptosis occurs).

### DNA damage and synapsis checkpoints eliminate germ cells that fail to form bivalents in *mrg-1*

**Fig 5:**
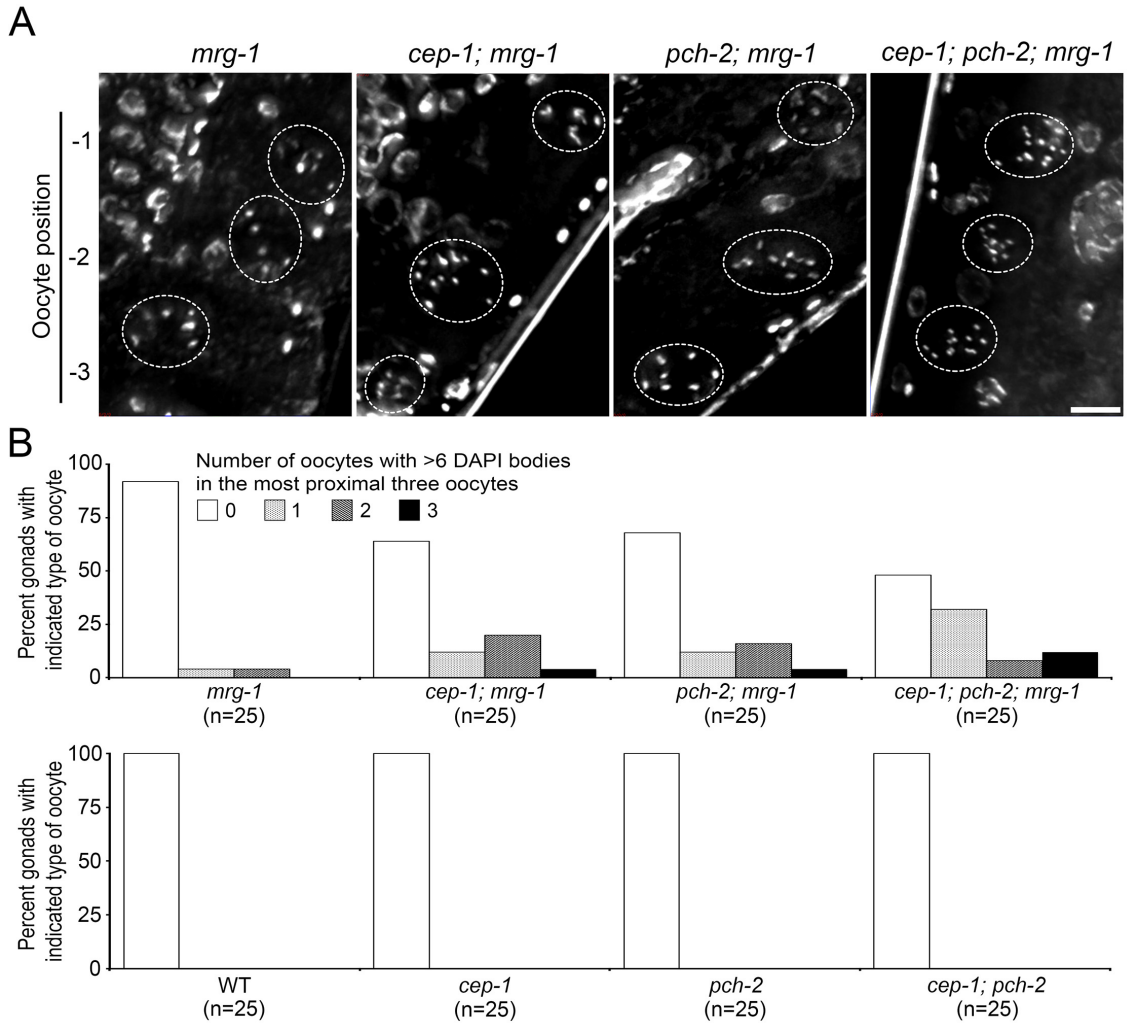
*mrg-1* (*lf*) exhibits increased failure to form bivalents when combined with meiotic checkpoint mutations (A) Full projections of optically sectioned DAPI-stained DNA images in oocytes from positions -1 to -3. The periphery of each oocyte is indicated by the dotted circle. Six DAPI-stained bodies per oocyte were usually observed in the *mrg-1* single mutant, demonstrating successful bivalent formation. The *cep-1; mrg-1* or *pch-2; mrg-1* double mutants frequently displayed diakinesis oocytes with more than six DAPI-stained bodies, indicating failed bivalent formation. The *cep-1; pch-2; mrg-1* triple mutant showed the most obvious failure in bivalent formation among the four strains tested. All four strains have ced-1::GFP in the background (not shown). The same image data that was analyzed in Fig 3 was used for this analysis. Bar: 10 μm. (B) Histogram showing the percentage of gonads with oocytes from the -1 to -3 positions displaying more than six DAPI-stained bodies. The *cep-1; mrg-1*, *pch-2; mrg-1*, and *cep-1; pch-2; mrg-1* mutants display substantially increased populations of oocytes with more than six DAPI-stained bodies in at least one of three late diakinesis oocytes. This increase is statistically significant except for *pch-2; mrg-1* when compared to the *mrg-1* single mutant (p = 0.0374, p = 0.0738, p = 0.0015, respectively, Fisher’s exact test).

The goal of meiotic quality control is to eliminate germ cells that fail to form bivalents by inducing germline apoptosis. Decreased germline apoptosis in *cep-1; mrg-1*, *pch-2; mrg-1* or *cep-1; pch-2; mrg-1* compared with *mrg-1* predicts that more diakinesis oocytes with univalents will be seen in these double or triple mutants. To directly test this prediction, we counted the number of DAPI-stained bodies in three oocytes from the proximal end (-1, -2 and, -3 positions) in these mutants. In the double mutant, *cep-1; mrg-1* or *pch-2; mrg-1*, 36% or 32% of the worms exhibited more than six DAPI-stained bodies in at least one of three late diakinesis oocytes, whereas only 8% of the *mrg-1* single mutant worms had such a phenotype (Fig 5). Thus, germ cells that fail to form bivalents in the *mrg-1* mutant are eliminated by germline apoptosis induced by DNA damage or synapsis checkpoints. Because, for this analysis, we used a randomly chosen subset of the image data sets that were used for apoptosis analysis, this result demonstrates a direct link between increased failure to form bivalents and decreased germline apoptosis due to *cep-1* or *pch-2* mutation. In the triple mutant, *cep-1; pch-2; mrg-1*, the population of worms exhibiting this phenotype increased to 52%, which is less than seen in the *ced-4 mrg-1* mutant (66%) where the core germline apoptosis machinery is defective. Although we failed to detect statistically significant differences between *cep-1; pch-2; mrg-1* and *ced-4 mrg-1*, this result is consistent with the possibility of a remaining activity to eliminate germ cells that fail to form bivalents in the triple mutant, *cep-1; pch-2; mrg-1*. This possibility is also consistent with our observation of increased germline apoptosis in the *cep-1; pch-2; mrg-1* triple mutant over control strains. This result further suggests that the remaining increase in germline apoptosis in the *cep-1; pch-2; mrg-1* triple mutant might be triggered by a novel meiotic checkpoint that eliminates part of the germ cells that fail to form bivalents independent of *cep-1* or *pch-2*.

## Materials and Methods

### Genetics

All C. *elegans* strains were cultured at 20°C under standard conditions [25]. The following strains and chromosome rearrangements were used: wild-type strain (Bristol N2 background); for Chromosome *I*, *cep-1(gk138)*; for Chromosome *II*, *pch-2(tm1458)*; for Chromosome III, *ced-4(n1162)*, *mrg1(qa6200)*, and *qC1*[*dpy-19(e1259) glp-1(q339) qls26] III*; for Chromosome *IV*, *ced-3(n717)*, *htp-1(gk174)*, *syp-1(me17)*, and *nT1[unc-*?*(n754) let-*? *Qls50](IV;V)*; for Chromosome V, *bcls39((P(lim-7)ced-1*::*gfp+lin-15(+))*.

### Cytological Analysis

#### Immunofluorescence

Gonad dissection, fixation for DAPI and antibody staining, and imaging were conducted as previously reported [26–28]. Worms were picked at the late L4 stage and cultured for 24 hours at 20°C before dissection. The following primary antibodies were used at the indicated dilutions: rabbit anti-HIM-3 [15], 1:1000; and guinea pig anti-SYP-1 [16], 1:1000. The following secondary antibodies were used at a dilution of 1:400: Alexa 488 anti-rabbit and Alexa 555 anti-guinea pig (Life Technologies). Images were obtained using a fluorescence microscope (BX61, Olympus) with a cooled CCD camera (ORCA-R2, Hamamatsu) controlled through cellSens Dimension software (Olympus). Optical sections were collected in three channels at 0.2-μm increments using a 100× oil objective (NA 1.30). Images were deconvolved with Huygens Essential software (Scientific Volume Imaging) using an iterative restorative algorithm for a maximum of 50 iterations.

#### Statistical analysis of scoring of bivalent formation

Fisher’s exact test (two-tailed) was used for statistical analysis to compare the distribution of gonads with oocytes featuring six DAPI-stained bodies, or gonads with oocytes featuring more than six DAPI-stained bodies between *mrg-1* and *ced-3; mrg-1*, *mrg-1* and *ced-4 mrg-1*, *mrg-1* and *ced-9; mrg-1*, *mrg-1* and *cep-1; mrg-1*, *mrg-1* and *pch-2; mrg-1*, or *mrg-1* and *cep-1; pch-2; mrg-1*.

#### Scoring of SC Assembly

Scoring for partial SC was conducted as previously reported [11]. For each strain, three gonads spanning the distal end to the pachytene exit were imaged in multiple frames. Beginning in the transition zone and moving toward the pachytene exit, all nuclei that were clearly resolved from one another were examined. Optical sections of each nucleus were analyzed to compare HIM-3 and SYP-1 staining. A nucleus with HIM-3 staining that did not overlap with SYP-1 staining was scored as partial SC if the following two conditions were satisfied: (1) the lack of SYP-1 staining was not due to lapses in continuous SYP-1 staining and (2) the surrounding area of partial synapsis showed strong SYP-1 staining. Gonad subzones were defined by subdivision into a pre-meiotic section consisting of the distal end of the gonad and extending to the transition zone, and the meiotic section containing the rest of the gonad. The total number of rows of nuclei (N) along the longitudinal axis of a gonad in the meiotic part was then determined, where the number of rows was counted on gonad images. N was then divided by 4, and the resulting value was rounded up (n). The width of the meiotic zone was then set to n rows of nuclei, except for the last row, which was set to N-3n rows of nuclei. The data from each gonad subzone were combined to determine the frequency of partial synapsis for SC assembly.

#### Imaging Apoptotic Nuclei

The ced-1::GFP transgene [19] was introduced into all strains examined. Worms were picked at the late L4 stage and cultured for 24 hours at 20°C. Worms were first desiccated with 95% ethanol, then treated with a 5 μg/mL DAPI solution for DNA staining. Images of the entire gonad were obtained using a fluorescence microscope (BX61, Olympus) with a cooled CCD camera (ORCA-r2, Hamamatsu) controlled through cellSens Dimension software (Olympus). Optical sections were collected in two channels with 0.6-μm increments using a 40× objective (NA 0.75). Images were then deconvolved with Huygens Essential software (Scientific Volume Imaging) using an iterative restorative algorithm for a maximum of 50 iterations.

#### Scoring of Apoptotic Cell Corpses

A minimum of 35 gonad arms was imaged for each strain. The exact number of gonad arms examined is shown in the figures. Subzones were defined by dividing gonads into two parts: one consisting of the region before the bend of the gonad arm, and another corresponding to the region after the bend of the gonad arm. The zone before the bend of the gonad arm, which spanned from the pre-meiotic region to the late pachytene and diplotene regions, was again divided into four subzones of equal length. The zone after the bend of the gonad arm, which contained the diplotene and diakinesis regions, was designated as a fifth subzone. All nuclei in these subzones were then examined for the presence of GFP signals from the CED-1::GFP transgene. Apoptotic cell corpses were defined as objects that satisfy three conditions: 1) retaining a nucleus stained with DAPI, 2) displaying a bright CED-1::GFP signal surrounding at least 75% of the periphery, and 3) having a signal from the surrounding CED-1::GFP of higher intensity than that surrounding non-apoptotic cells (i.e., background fluorescence). Apoptotic cell corpses in each subzone were then counted. For statistical analysis, the Mann-Whitney U test (two-tailed) was used for statistical analysis to compare the mean number of total apoptotic cell corpses for each gonad between each strain.
